# Comparison between Organic and Inorganic Zinc Forms and Their Combinations with Various Dietary Fibers in Respect of the Effects on Electrolyte Concentrations and Mucosa in the Large Intestine of Pigs

**DOI:** 10.3390/ijms242316743

**Published:** 2023-11-25

**Authors:** Marcin Barszcz, Kamil Gawin, Anna Tuśnio, Adrianna Konopka, Ewa Święch, Marcin Taciak, Jacek Skomiał, Katarina Tokarčiková, Klaudia Čobanová, Ľubomira Grešáková

**Affiliations:** 1 Department of Animal Nutrition, The Kielanowski Institute of Animal Physiology and Nutrition, Polish Academy of Sciences, Instytucka 3, 05-110 Jabłonna, Poland; k.gawin@ifzz.pl (K.G.); a.tusnio@ifzz.pl (A.T.); a.konopka@ifzz.pl (A.K.); e.swiech@ifzz.pl (E.Ś.); j.skomial@ifzz.pl (J.S.); 2 Division of Animal Nutrition, Institute of Animal Sciences, Warsaw University of Life Sciences, Ciszewskiego 8, 02-786 Warsaw, Poland; marcin_taciak@sggw.edu.pl; 3 Institute of Animal Physiology, Centre of Biosciences of the Slovak Academy of Sciences, Soltesovej 4, 04001 Kosice, Slovakia; tokarcikova@saske.sk (K.T.); boldik@saske.sk (K.Č.); gresakl@saske.sk (Ľ.G.); 4 Department of Animal Morphology, Physiology and Genetics, Faculty of AgriSciences, Mendel University in Brno, 613 00 Brno, Czech Republic

**Keywords:** zinc, potato fiber, electrolytes, mucins, mucosa, colon, piglet

## Abstract

This study aimed to determine the effects of Zn sources, used with potato fiber (PF) or lignocellulose (LC), on electrolyte concentration and the mucus layer in the large intestine of pigs. The experiment involved 24 barrows with an initial body weight of 10.8 ± 0.82 kg, divided into four groups fed the following diets: LC and ZnSO_4_, LC and Zn glycinate (ZnGly), PF and ZnSO_4_, or PF and ZnGly. Fiber supplements provided 10 g crude fiber/kg diet, while Zn additives introduced 120 mg Zn/kg diet. After four weeks of feeding, the pigs were sacrificed and digesta and tissue samples were taken from the cecum and colon. PF increased the water content and decreased the phosphorus concentration in the large intestine in comparison with LC. PF also increased calcium, iron, and chloride concentrations in the descending colon. Mucus layer thickness and histological parameters of the large intestine were not affected. ZnGly diets increased *MUC12* expression in the cecum as compared to the LC-ZnSO_4_ group. In the ascending colon, the PF-ZnGly diet increased *MUC5AC* expression, while both PF groups had greater *MUC20* expression in comparison with the LC-ZnSO_4_ group. In the transverse colon, the LC-ZnGly group and both PF groups had higher *MUC5AC* expression in comparison with the LC-ZnSO_4_ group, and both ZnGly groups had higher *MUC20* expression than ZnSO_4_ groups. PF and ZnGly increased *MUC4* and *MUC5AC* expression in the descending colon. PF and ZnGly may exert a beneficial effect on colon health in pigs by upregulating the expression of the *MUC5AC* and *MUC20* genes and are more effective than LC and ZnSO_4_.

## 1. Introduction

The main functions of the large intestine are the reabsorption of water and electrolytes secreted into the gut lumen during digestion, the excretion of toxic substances and waste products of metabolism, and the provision of a habitat for the growth and development of the complex microbiota population that participates in the salvation of energy and nutrients through fermentation [1]. Absorptive colonocytes are the predominating cell population in the epithelium [2], which constitutes the border between the gut lumen and the internal environment of the body. The epithelium is in permanent contact with bacteria and chemical compounds of exo- and endogenous origin, and this contact is mediated by two layers of mucus secreted by goblet cells, i.e., a loose outer layer and an inner layer that is firmly attached [3,4]. Mucus belongs to the innate immunity system and constitutes a physical barrier for pathogens and other detrimental factors of endo- and exogenous origin. It binds bacterial adhesins, maintains high concentrations of secretory immunoglobulin A and lysozyme on the surface of the epithelium, and removes free radicals. The mucus layer participates in the transport of substances between the gut lumen and epithelial cells, lubricates the mucosal surface, which facilitates digesta passage, protects the epithelium from excessive mechanical stress, and ensures a suitable environment for intestinal microbiota [5]. The main constituent of mucus, responsible for its physiological properties, are mucins, which are filamentous glycoproteins of high molecular weight, which can exceed 100 MDa [6]. Mucins are characterized by a peptide backbone rich in proline, threonine, and serine. Threonine and serine residues are binding sites for *O*-glycans, which form oligosaccharide side chains that comprise over 70% of mucin weight and are responsible for their bottle-brush-like conformation [6,7].

Mucins can be divided into three subfamilies and are encoded by many genes. Secreted gel-forming mucins are encoded by the *MUC2*, *MUC5AC*, *MUC5B*, *MUC6,* and *MUC19* genes, while secreted non-gel-forming mucins are encoded by *MUC7* and *MUC8*. Genes of cell surface mucins are *MUC1*, *MUC3A/B*, *MUC4*, *MUC12*, *MUC13*, *MUC15*, *MUC16*, *MUC17*, and *MUC20* [7]. Gel-forming mucins are major constituents of mucus and are responsible for its properties, while membrane-associated mucins are important components of the glycocalyx, which may limit the access of other cells and large molecules to the cell surface and may be involved in signal transduction [7,8]. Regarding the diet, its effect on *MUC1*, *MUC2*, *MUC13*, and *MUC20* expression has been most often studied in pigs [9,10,11,12,13,14,15].

Zn is a trace mineral necessary for the appropriate function of the intestinal barrier. It was demonstrated that Zn content ranging from 57 to 2425 mg/kg diet did not affect *MUC1*, *MUC2*, *MUC13*, or *MUC20* expression in the ascending colon when ZnO was used as the supplement [11]. On the other hand, in vitro research on goblet cells showed that Zn deficiency increased *MUC2* and tended to decrease *MUC5AC* expression, as well as impaired *O*-glycosylation, leading to the synthesis of shorter oligosaccharide side chains and the modification of the *O*-glycan pattern [16]. Data concerning the effect of inorganic and organic Zn sources on mucin genes are scarce. One such study demonstrated that *MUC1* and *MUC2* expression in the jejunum was similar in weaned piglets fed diets supplemented either with 100 mg/kg Zn glycinate (ZnGly) or ZnSO_4_ [17]. So far, differences between Zn sources in regard to mucosal physiology in the large intestine have not been investigated.

Another important factor affecting the intestinal immune barrier is the soluble dietary fiber pectin. This polysaccharide mainly consists of linear 1,4-D-galacturonan segments and branched rhamnogalacturonan segments, and can be found in apples, citrus fruits, sugar beets, and potatoes [18,19]. Pectins protect the intestinal barrier from damage via direct interaction with immune receptors, stimulation of intestinal microbiota populations, strengthening of the mucus layer, and stimulation of mucin secretion [18]. These complex carbohydrates may also interact with minerals affecting their absorption in the small intestine. However, after microbial breakdown in the large intestine, nutrients that were trapped by polysaccharides become available for the host or microbiota [20,21].

Previously, it was found that potato fiber (PF), which is rich in pectin, cellulose, hemicelluloses, and starch, increased the digestibility of crude fiber, detergent fiber fractions, total phosphorus, and Zn in pigs in comparison with lignocellulose (LC) [22]. In the same study, it was discovered that the digestibility of crude ash and Zn was lower, while that of acid detergent fiber, cellulose, and starch was higher in pigs fed diets supplemented with ZnGly than in those given ZnSO_4_. Further research revealed that the interaction between Zn source and fiber type affected *Clostridium* spp. populations in the cecum and middle colon, concentration of ammonia and activity of bacterial β-glucosidase in the proximal colon, and isoacid concentration in the distal colon. Independently of Zn source, PF improved dietary fiber digestibility by increasing β-glucosidase activity, while ZnGly, regardless of fiber supplement, stimulated the growth of *Clostridium herbivorans* and increased total phenols concentration [23]. These findings clearly show that PF and ZnGly change the large intestine environment, which may affect the mucosa. Therefore, in a continuation of the research, the hypothesis was that ZnGly and PF influence electrolyte concentrations and beneficially affect the large intestine mucosa by modulation of mucin gene expression and improvement of histological parameters. The research goal was to determine the effects of Zn source, used with PF or LC, on electrolyte concentrations, mucus layer thickness, mucin gene expression, and histological parameters in the cecum and colon of pigs.

## 2. Results

### 2.1. Water and Electrolyte Content

Feeding PF diets significantly increased water content in the cecum (*p* = 0.039), ascending colon (*p* = 0.039), and descending colon (*p* = 0.002) of pigs in comparison with LC diets. There was also a tendency toward greater water content in the transverse colons of animals fed PF diets. In each part of the large intestine, there was an effect of fiber on inorganic phosphate concentrations, which were lower in pigs fed PF diets as compared to animals on LC diets (*p* < 0.05). Fiber type affected also sodium and calcium concentrations in the transverse colon, where they were greater in pigs given PF diets (*p* = 0.020 and *p* = 0.037, respectively). A similar effect of PF was found in the descending colon in the case of calcium (*p* = 0.009), iron (*p* = 0.001), and chloride (*p* = 0.041) concentrations (Table 1). Neither the Zn source nor the interaction between experimental factors affected water and electrolyte contents in the large intestine digesta of pigs.

### 2.2. Mucus Layer Thickness and Large Intestine Morphology

**Figure 1:**
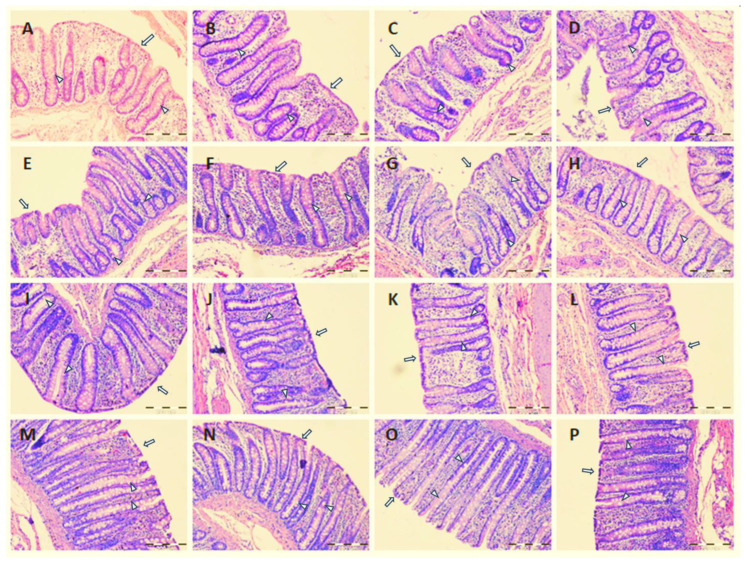
Representative images of hematoxylin–eosin-stained sections of the cecum (**A**–**D**), ascending colon (**E**–**H**), transverse colon (**I**–**L**), and descending colon (**M**–**P**) of pigs fed diets supplemented with LC and ZnSO_4_ (**A**,**E**,**I**,**M**), LC and ZnGly (**B**,**F**,**J**,**N**), PF and ZnSO_4_ (**C**,**G**,**K**,**O**), and PF and ZnGly (**D**,**H**,**L**,**P**). White arrows indicate an intact layer of absorptive colonocytes, while white arrowheads indicate goblet cells. Scale bar—200 μm.

There was no effect of experimental factors on mucus layer thickness, crypt depth, and muscular layer thickness in the large intestine of pigs. Only tendencies toward a reduction in crypt depth were noticed in the cecum and ascending colon of pigs fed PF diets (*p* = 0.063 and *p* = 0.094, respectively). There were no statistical differences between ZnSO_4_ and ZnGly as well as between LC and PF diets in regard to intraepithelial lymphocyte count (Table 2). Feeding experimental diets did not cause mucosal damage in any segment of the large intestine of pigs (Figure 1).

### 2.3. Mucin Gene Expression

**Figure 2:**
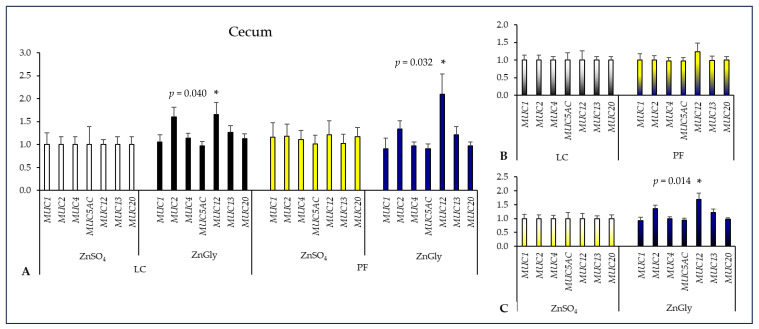
The effect of dietary treatments on mucin gene expression in the cecum of pigs. LC—lignocellulose, PF—potato fiber, ZnGly—zinc chelate of glycine hydrate. Asterisks indicate means that differ significantly from the control group at *p* < 0.05. (**A**) Gene expression was calculated considering pigs fed a diet with LC and ZnSO_4_ as the control group (expression = 1). (**B**) Gene expression was calculated considering both LC groups as the control (expression = 1) to compare dietary fiber supplements. (**C**) Gene expression was calculated considering both ZnSO_4_ groups as the control (expression = 1) to compare Zn additives.

Both groups of pigs fed diets supplemented with ZnGly had significantly higher *MUC12* expression in the cecum as compared to the control group fed a diet supplemented with LC and ZnSO_4_ (*p* < 0.05). There was also a significant difference between Zn sources regarding *MUC12* expression, which was greater (*p* = 0.014) in pigs fed ZnGly diets than in those fed ZnSO_4_ diets. There were no differences in the expression of mucin genes between pigs fed LC and PF diets (Figure 2).

**Figure 3:**
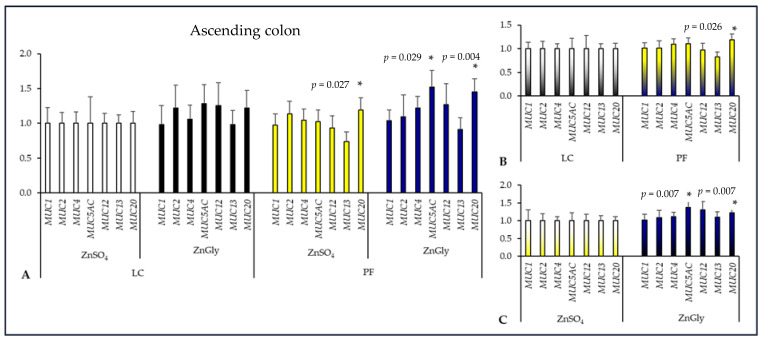
The effect of dietary treatments on mucin gene expression in the ascending colon of pigs. LC—lignocellulose, PF—potato fiber, ZnGly—zinc chelate of glycine hydrate. Asterisks indicate means that differ significantly from the control group at *p* < 0.05. (**A**) Gene expression was calculated considering pigs fed a diet with LC and ZnSO_4_ as the control group (expression = 1). (**B**) Gene expression was calculated considering both LC groups as the control (expression = 1) to compare dietary fiber supplements. (**C**) Gene expression was calculated considering both ZnSO_4_ groups as the control (expression = 1) to compare Zn additives.

In the ascending colon (Figure 3), pigs receiving a diet with PF and ZnGly had significantly higher *MUC5AC* expression (*p* = 0.029), while both PF groups had greater *MUC20* expression in comparison with the control group (*p* < 0.05). There was also a difference between fiber supplements in regard to *MUC20* expression, which was higher in pigs fed diets supplemented with PF than in those fed LC diets (*p* = 0.026). Pigs fed ZnGly diets had significantly greater expression of *MUC5AC* and *MUC20* in comparison with those given ZnSO_4_ diets (*p* < 0.01).

**Figure 4:**
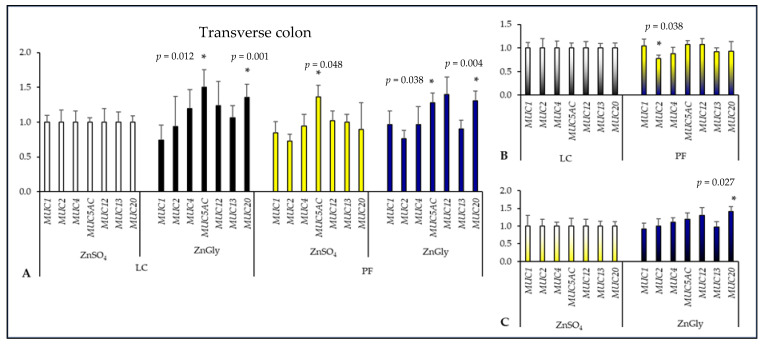
The effect of dietary treatments on mucin gene expression in the transverse colon of pigs. LC—lignocellulose, PF—potato fiber, ZnGly—zinc chelate of glycine hydrate. Asterisks indicate means that differ significantly from the control group at *p* < 0.05. (**A**) Gene expression was calculated considering pigs fed a diet with LC and ZnSO_4_ as the control group (expression = 1). (**B**) Gene expression was calculated considering both LC groups as the control (expression = 1) to compare dietary fiber supplements. (**C**) Gene expression was calculated considering both ZnSO_4_ groups as the control (expression = 1) to compare Zn additives.

In the transverse colon (Figure 4), there was no effect of experimental factors on *MUC1*, *MUC4*, *MUC12*, and *MUC13* expression but pigs fed an LC diet with ZnGly and both PF groups had higher *MUC5AC* expression in comparison with the control group (*p* < 0.05). Feeding diets supplemented with PF significantly reduced the expression of *MUC2* as compared to LC diets (*p* = 0.038), while ZnGly supplementation increased *MUC20* expression in comparison with ZnSO_4_ (*p* = 0.027). There were no other differences between Zn and fiber sources in this segment of the large intestine in regard to mucin gene expression.

**Figure 5:**
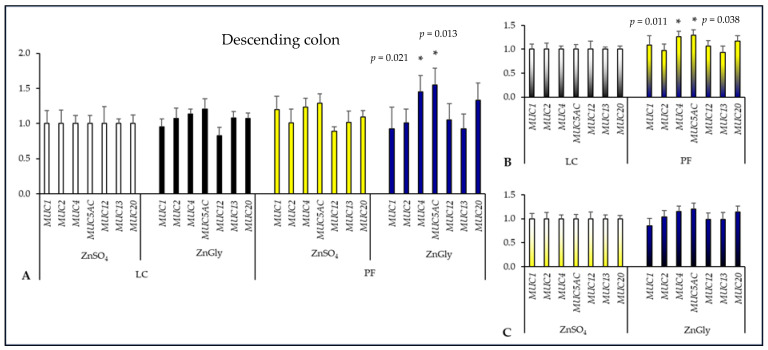
The effect of dietary treatments on mucin gene expression in the descending colon of pigs. LC—lignocellulose, PF—potato fiber, ZnGly—zinc chelate of glycine hydrate. Asterisks indicate means that differ significantly from the control group at *p* < 0.05. (**A**) Gene expression was calculated considering pigs fed a diet with LC and ZnSO_4_ as the control group (expression = 1). (**B**) Gene expression was calculated considering both LC groups as the control (expression = 1) to compare dietary fiber supplements. (**C**) Gene expression was calculated considering both ZnSO_4_ groups as the control (expression = 1) to compare Zn additives.

Feeding pigs a diet supplemented with PF and ZnGly significantly increased *MUC4* and *MUC5AC* expression in the descending colon in comparison with the control group (*p* = 0.021 and *p* = 0.013, respectively). There were no differences in mucin gene expression between pigs fed the LC diet with ZnSO_4_ and those given the LC diet with ZnGly and the PF diet with ZnSO_4_. Pigs fed diets with the addition of PF had significantly higher *MUC4* and *MUC5AC* expression than animals on LC diets (*p* = 0.011 and *p* = 0.038, respectively). The expression of mucin genes in the descending colon did not differ between pigs fed ZnGly and ZnSO_4_ diets (Figure 5).

## 3. Discussion

To the best of our knowledge, there have been no studies regarding the interactive effect of Zn and dietary fiber supplementation on the large intestine epithelium. Since one of its most important roles is the reabsorption of water and electrolytes, their concentrations in the digesta serve as a valuable marker of mucosa cell function. A higher concentration of sodium and potassium indicates an impaired reabsorption capacity and is associated with increased damage of the surface epithelium and a decreased amount of the host DNA detected in feces, which is a marker of exfoliated epithelial cells [24]. In the colon, chloride absorption occurs mainly via the electroneutral exchange pathway and is coupled with sodium absorption [25]. Therefore, it may be also used as a marker of colonocyte function. In the present study, pigs fed PF diets had greater water content in all parts of the large intestine (tendency in the transverse colon) than pigs fed LC diets, though the differences were small. This effect could have resulted from a high content, i.e., 45%, of soluble fiber responsible for high water binding and swelling capacity of PF [26], which may be slightly higher than those of LC. The main constituent of the soluble fiber fraction of PF is pectin, while that of insoluble fiber is cellulose [26]. Due to their chemical structure, these polysaccharides may affect mineral absorption [20], which, in the current research, was shown to be the case for sodium, calcium, iron, and chloride, particularly in the descending part of the pig colon. Other authors [27] showed that dietary supplementation with different fibers such as apple pomace, PF, and sugar beet pulp increased fecal excretion of calcium, magnesium, iron, manganese, zinc, and copper in rats and decreased the apparent absorption of these minerals. This is only in partial agreement with the results of the current research, because feeding PF diets increased the apparent digestibility of Zn and had no effect on that of crude ash in the same animals, as was described previously [22]. Higher concentrations of sodium, calcium, iron, and chloride in the descending colon of pigs fed PF-supplemented diets probably resulted from the microbial breakdown of pectin, which caused a release of these ions to fecal water, as suggested by other authors [20,21]. It is also known that epithelial ion transport is affected by some inflammatory cytokines. Tumor necrosis factor-α and interleukin-1β stimulate chloride secretion, while interleukin-1α inhibits sodium and chloride absorption [28]. Unfortunately, these cytokines were not the subject of investigation in the current study, but there were no signs of inflammation, e.g., neutrophil infiltration, in the pigs’ mucosa. The number of intraepithelial lymphocytes, which may facilitate electrolyte transport across the epithelium [29], also did not differ between experimental groups and was similar to that found in the cecum and colon of healthy pigs in other studies [30,31].

In the case of calcium, a higher concentration in the digesta may exert an anti-proliferative effect in the colon by precipitating surfactants like fatty acids and bile acids, thus reducing their cytotoxicity. The beneficial effect of calcium on the integrity of the intestinal epithelium is associated with a decreased activity of intestinal alkaline phosphatase in the fecal water, which is a marker of intestinal epitheliolysis [32,33]. This effect of PF may be of importance when considering a pig as a model animal for humans. The distal colon is the main site of colon cancer development [34]. Therefore, diets that favor a higher calcium concentration in the large intestine may be beneficial for health status. On the other hand, feeding PF diets increased the iron concentration in the descending colon. This is in line with the results of Tokarčíková et al. [35], who found a reduced iron digestibility in the small intestine and in the whole digestive tract of the same pigs fed PF diets. Analyzing free iron concentration in the large intestine is also important from the point of view of colorectal cancer development [36]. Dietary iron, unabsorbed in the small intestine, may enter the cecum and colon and participate in Fenton reactions, which increases the production of hydrogen peroxide and hydroxyl radicals at the surface of the mucosa [37]. Hydrogen peroxide and iron may increase the risk of DNA damage and mutations in colonocytes. Iron may be also involved in the conversion of procarcinogens to carcinogens in the lumen of the large intestine [37]; therefore, it is regarded as a tumor promoter [36]. Studies on rats showed that a high-iron diet (102 mg/kg diet) increased intraluminal iron concentration and exerted a hyperproliferative effect on cecal and colonic crypts [36]. Iron is also one of the micronutrients essential for the growth and virulence of most pathogenic bacteria [38]. Considering that feeding PF diets had no effect on crypt depth in the large intestine of pigs as compared to LC diets, it may be assumed that the proliferation rate of colonocytes was unaffected by higher concentrations of calcium and iron.

In contrast to calcium, the concentration of magnesium was unaffected by dietary treatments in any segment of the large intestine of pigs. A lower concentration of magnesium in the lumen serves as an indicator of reduced damage of epithelial cells because they contain relatively large amounts of this mineral [39,40,41]. Feeding pigs PF and ZnGly diets did not affect magnesium concentrations in the large intestine in comparison with LC and ZnSO_4_ diets, respectively, which is in line with the results of histological examination, which showed no mucosal damage.

Inorganic phosphate concentration in the large intestine digesta depends on many factors, including total and phytate phosphorus concentration in the diet, phytase and alkaline phosphatase activities in the gut [23,42], absorption of phosphorus in the small intestine [25], digestibility [22], secretion into the gut lumen, the source of fermentable carbohydrates, and incorporation into microbial biomass [43]. Feeding diets differing in Zn source had no effect, while PF diets reduced inorganic phosphorus concentration in all parts of the large intestine, which mirrors the increased total tract apparent digestibility of total phosphorus observed earlier in the same pigs [22]. Since there was no effect of dietary treatments on phytase and alkaline phosphatase in the large intestinal digesta of these pigs [23], their effect on phosphorus content may be explained by an improved solubility of mineral complexes in the gut due to feeding PF-supplemented diets.

The mucus layer undergoes continuous degradation and renewal, and changes in its properties may influence the absorption of nutrients, endogenous macromolecules, and electrolytes [44,45]. In the present study, there was no effect on mucus adherent layer thickness in the large intestine, which was measured using the spectrophotometric method. This result is in agreement with previous research on the same pigs, which showed no differences in the activity of bacterial mucinase [23], which is used both by pathogens and commensal microbiota to break down the mucus [46,47,48]. Despite the unaffected mucus layer thickness, there were some changes in the expression of mucin genes evoked by the dietary treatments. Feeding LC and PF diets supplemented with ZnGly increased the expression of *MUC12* in the cecum but not in the colon of pigs. This mucin belongs to the type of cell surface mucin [7]. The limitation of expression changes to the cecum suggests that it may be related to other diet-induced changes in this part of the large intestine. The same diets that enhanced *MUC12* expression reduced propionate concentration, increased the relative abundance of *C. herbivorans*, and tended to decrease that of *Lactobacillus* spp. [23]. However, further studies in larger numbers of pigs are needed to determine if there are correlations between these variables. The lack of effect of ZnGly on *MUC12* expression in the proximal colon, where *C. herbivorans* population was also greater as compared to ZnSO_4_ diets, suggests that propionate concentration and *Lactobacillus* spp. abundance might be of greater importance.

In the current study, it was found that *MUC5AC* expression was affected by the experimental factors in each segment of the colon, *MUC20* expression was affected in the ascending and transverse parts, while *MUC2* and *MUC4* expression was affected only in the transverse and descending segment, respectively. A higher expression of *MUC5AC* and *MUC20* in pigs fed PF diets with ZnGly than in those fed LC diets with ZnSO_4_ suggests an improvement of the protective barrier in the colon. The fact that the expression of these two mucin genes was also enhanced in the transverse colon of pigs fed an LC diet with ZnGly suggests that the addition of an organic Zn form to a diet supplemented with insoluble dietary fiber may be beneficial for the health status of the colon. The effects of dietary treatments on mucin gene expressions were not uniform but differed between segments of the large intestine of pigs. This might result from differences in microbiota composition and activity. Along the large intestine, concentrations of the main short-chain fatty acids (SCFAs), i.e., acetic, propionic, and butyric acids, decrease, while those of branched-chain fatty acids (iso-valeric, iso-butyric), ammonia, amines, and phenolic and indolic compounds increase due to the depletion of carbohydrates for fermentation and more intensive proteolysis [23,49,50]. Also, the activity of bacterial enzymes (β-glucuronidase, β-glucosidase, mucinase) and the relative abundance of bacterial populations change along the large intestine of pigs [23,30,31]. These factors affect the health status of the mucosa and may contribute to differences in the effects of diets on the expression of mucin genes found between different parts of the large intestine. Nonetheless, further research is required to determine all factors regulating *MUC* gene expressions.

Zhou et al. [51] showed that a long-term intake of a diet with high resistant starch content upregulated the expression of *MUC4*, *MUC5AC*, and *MUC12*, and reduced proteolytic fermentation in the colon of pigs. Since PF is not a pure source of dietary fiber but also contains ca. 24% starch [22], the upregulated expression of *MUC4* and *MUC5AC* in the descending colon of pigs fed PF diets may be partially explained by the effect of potato starch. The beneficial effect of PF might also result from the presence of pectins, which strengthen the mucus layer and stimulate mucin secretion [18]. Furthermore, it should be noted that ZnGly supplementation enhanced the effect of PF.

Other authors [52] suggested that bacterial infection leads to increased *MUC5AC* expression in the colon. It is thought that this mucin may play a role in the adhesion of *Salmonella typhimurium* to the colonic epithelium and that its upregulation, accompanied by trefoil factor 1, may occur in response to barrier damage and have a role in tissue repair [52]. However, in the current study, pigs remained healthy throughout the whole experimental period and no signs of infection were observed. Thus, the upregulation of *MUC5AC* expression observed in the colon of pigs fed a diet supplemented with PF and ZnGly rather indicates improved gut health.

The exact mechanisms of action of Zn and fiber on mucin gene expression are largely unknown. In vitro research demonstrated that SCFAs stimulate the expression of *MUC2* through the mitogen-activated protein kinase signaling pathway [53] and that histone modifications and the AP-1 transcription factor are involved in the regulation of *MUC2* [54]. The protein encoded by this gene is the major mucin produced in the small and large intestine, belongs to the class of secretory gel-forming mucins [3], and forms the skeleton of the two mucus layers [6]. Previous evidence indicated that stimulation of *MUC2* expression by SCFAs was mediated by prostaglandins produced by subepithelial myofibroblasts and epithelial cells. SCFAs increased the production of prostaglandin E_1_ and reduced that of prostaglandin E_2_ by myofibroblasts, and prostaglandin E_1_ was found to be superior to E_2_ in the upregulation of *MUC2* expression [55]. However, there were no effects of dietary treatments on the concentrations of acetate, propionate, and butyrate in the colon of these pigs, which was described earlier [23]. Moreover, feeding with PF diets contributed to a reduction of *MUC2* expression in the middle colon. Therefore, it remains to be elucidated whether the supplementation of a diet with PF affects prostaglandin production and other mechanisms involved in the regulation of *MUC2* expression. Considering that different mucin genes differ in their response to dietary treatments, further research is needed to explain the mechanisms responsible for the regulation of their expression.

## 4. Materials and Methods

### 4.1. Animals and Diets

The design of this study and housing conditions were described previously [22,56]. The two-factorial experiment involved 24 forty-day-old Danbred x Duroc barrows with an initial body weight of 10.8 ± 0.82 kg. Pigs were divided into four groups (*n* = 6) fed cereal-based diets supplemented with: 1.7% LC and 0.033% ZnSO_4_, 1.7% LC and 0.046% ZnGly, 5% PF and 0.033% ZnSO_4_, and 5% PF and 0.046% ZnGly. LC and PF introduced 10 g crude fiber/kg to each diet, while ZnSO_4_ and ZnGly introduced 120 mg Zn/kg. The Zn supplements used in the experiment were ZnSO_4_ monohydrate (Sigma-Aldrich Corp., Saint Louis, MO, USA) and Zn chelate of glycine hydrate (Glycinoplex-Zn 26%, Phytobiotics Futterzusatzstoffe GmbH, Eltville, Germany), whereas fiber supplements included LC (Lonocel, Cargill Poland Ltd., Kiszkowo, Poland) and PF (Potex, Lyckeby Starch, Kristianstad, Sweden). The content of the vitamin–mineral premix was adjusted in each diet to 0.4% by the addition of Zn supplement. Diets were isoenergetic and isoprotein, and contained 4% crude fiber and 142 mg Zn/kg. The ingredients and chemical compositions of the experimental diets were described previously [22].

Pigs were kept individually in pens with free access to feed and water throughout the whole four-week-long experimental period. At the end of the trial, pigs were stunned by electric shock and sacrificed by exsanguination. The large intestine was removed to collect tissue and digesta samples from the cecum (ca. 7 cm from the ileo-cecal-colonic junction towards the bottom of the cecum), ascending colon (15 cm from the ileo-cecal-colonic junction towards the anus), transverse colon (at the half length of the entire colon), and descending colon (50 cm before the anus).

### 4.2. Analyses of Water Content and Electrolyte Concentrations

Water content in intestinal digesta was analyzed by the standard gravimetric method according to AOAC procedure no. 934.01 [57]. To determine electrolyte concentration, digesta samples (ca. 1.0 g) were homogenized in 2 mL (for sodium and potassium) or 3 mL (for other ions) of ultrapure water (18.2 MΩ) for 30 s at high speed. After centrifugation (12,850× *g*, 10 min, room temperature), supernatants were taken and Na and K concentrations were measured using an EasyLyte Na/K Analyzer (Medica, Bedford, MA, USA). Calcium, magnesium, iron, chloride, and inorganic phosphorus concentrations were measured spectrophotometrically using a Maxmat PL biochemical analyzer (Erba Diagnostics France SARL, Montpellier, France) and suitable diagnostic reagents (ELITechGroup Clinical Systems, Puteaux, France).

### 4.3. Measurement of Mucus Layer Thickness

Cecum and colon samples were placed in ice-cold Krebs–Henselait buffer saturated with carbogen and kept on ice until the end of sampling. Then, 1 × 1 cm pieces were cut in triplicate, and mucus layer thickness was measured spectrophotometrically according to Smirnov et al. [44] and expressed in μg alcian blue dye absorbed by 1 cm^2^ of tissue.

### 4.4. Histological Examination of the Large Intestine

Paraffin-embedded pieces of cecum and colon were sliced into 4.5 µm sections using a rotary microtome HM355S (Thermo Shandon Limited, Runcorn, UK). Then, the slides were deparaffinized with xylene, hydrated through graded ethanol to water, and stained with hematoxylin and eosin. Crypt depth and muscular layer thickness (10 measurements per animal) were determined using a Zeiss Axio Star Plus (Carl Zeiss, Göttingen, Germany) light microscope and Axio Vision LE Rel. 4.5 (Carl Zeiss, 2002–2005) image analysis software. Intraepithelial lymphocytes were counted at 40× objective using an Olympus BX51 microscope (Olympus Corp., Tokyo, Japan) and Cell^D^ Imaging Software ver. 2.4.112-240608 (Olympus Soft Imaging Solutions GmbH, Munster, Germany). For each sample, 500–600 epithelial cells and all lymphocytes between them were counted.

### 4.5. Total RNA Isolation

Total RNA was isolated from the cecum and colon samples using an EXTRACTME Total RNA Kit (BLIRT SA DNA-Gdansk Division, Gdańsk, Poland). RNA integrity was checked by electrophoresis on 2% agarose gel stained with ethidium bromide, while its concentration and purity were determined on a NanoDrop ND-1000 spectrophotometer (Thermo-Scientific, Wilmington, DE, USA).

### 4.6. Gene Expression Analysis

Expressions of mucin genes (*MUC1*, *MUC2*, *MUC4*, *MUC5AC*, *MUC12*, *MUC13*, *MUC20*) and the *GAPDH* gene were measured by the quantitative real-time PCR method. Reverse transcription and cDNA synthesis were performed using the TRANSCRIPTME RNA kit (BLIRT SA DNA-Gdansk Division). Primers used for the amplification were designed according to sequences published previously [9] and synthesized by BLIRT SA DNA-Gdansk Division. For each reaction, 1 μL of cDNA, 5 μL of 2× AMPLIFYME SG Mix (BLIRT SA DNA-Gdansk Division), and 0.3 μL of each primer were added and made up to a final volume of 10 μL using nuclease-free water. Reactions were carried out using a MIC qPCR thermocycler (Bio Molecular Systems, Upper Coomera, Australia) according to the following program: one cycle of 95 °C for 3 min and 40 cycles of 95 °C for 5 s, 60 °C for 10 s, and 72 °C for 15 s. Relative gene expression was calculated from the cycle threshold value (Ct) using the 2^−ΔΔCt^ method [58] and *GAPDH* as the reference gene. The calculations of gene expression were performed considering a group of pigs fed a diet with LC and ZnSO_4_ as the control group (expression = 1). Additional calculations were performed to compare Zn sources (ZnSO_4_ groups as the control) and fiber supplements (LC groups as the control).

### 4.7. Statistical Analysis

Data were analyzed by two-way ANOVA followed by Fisher’s least significant difference post hoc test using the Statgraphics Centurion XVI ver. 16.1.03 statistical package (StatPoint Technologies, Inc., Warrenton, VA, USA). The normality assumption for ANOVA was checked by the Shapiro–Wilk test. The results of qPCR were analyzed with a two-tailed Student’s *t*-test using micPCR software 2.10 (Bio Molecular Systems, Upper Coomera, Australia). The level of significance was set at *p* ≤ 0.05.

## 5. Conclusions

Feeding PF diets increases the water content and reduces the phosphorus concentration in the large intestine digesta in pigs. It also increases calcium, iron, and chloride concentrations in the descending colon. Neither PF nor ZnGly affect histological parameters and mucus layer thickness. However, both supplements may exert beneficial effects on colon health in pigs by upregulating the expression of the *MUC5AC* and *MUC20* genes and are more effective than LC and ZnSO_4_.

## Figures and Tables

**Table 1:** Water and electrolyte content in the large intestine digesta of pigs.

Parameter	LC		PF		SEM	P		
	ZnSO_4_	ZnGly	ZnSO_4_	ZnGly		Fiber	Zn	Interaction
Cecum								
Water, %	86.34	87.50	88.26	88.23	0.324	0.039	0.360	0.334
Na, mmol/g	0.130	0.131	0.131	0.132	0.0014	0.769	0.894	0.979
K, mmol/g	0.015	0.011	0.014	0.013	0.0006	0.942	0.079	0.347
Ca, μmol/g	3.78	2.82	3.82	3.34	0.261	0.598	0.188	0.659
Fe, μmol/g	0.052	0.054	0.057	0.049	0.002	0.949	0.566	0.342
Cl, μmol/g	17.87	18.41	22.16	17.64	0.848	0.292	0.234	0.135
Mg, μmol/g	8.13	7.01	7.23	6.92	0.330	0.475	0.302	0.554
P, μmol/g	11.46	10.06	9.68	6.88	0.590	0.025	0.053	0.504
Ascending colon								
Water, %	82.62	83.69	84.10	84.96	0.339	0.039	0.139	0.865
Na, mmol/g	0.126	0.131	0.129	0.132	0.0014	0.590	0.209	0.670
K, mmol/g	0.023	0.019	0.019	0.020	0.0006	0.238	0.300	0.046
Ca, μmol/g	3.86	3.24	4.20	4.08	0.198	0.150	0.359	0.534
Fe, μmol/g	0.072	0.072	0.075	0.072	0.003	0.775	0.813	0.852
Cl, μmol/g	20.03	18.84	22.27	20.00	0.691	0.231	0.222	0.700
Mg, μmol/g	10.92	9.94	9.65	10.24	0.311	0.449	0.760	0.228
P, μmol/g	17.01	16.18	13.94	12.24	0.688	0.009	0.309	0.723
Transverse colon								
Water, %	77.46	77.75	80.54	78.76	0.527	0.053	0.462	0.312
Na, mmol/g	0.108	0.116	0.123	0.120	0.0020	0.020	0.494	0.123
K, mmol/g	0.037	0.032	0.031	0.032	0.0016	0.311	0.592	0.410
Ca, μmol/g	3.01	2.37	3.71	4.06	0.283	0.037	0.787	0.363
Fe, μmol/g	0.090	0.101	0.092	0.108	0.003	0.471	0.056	0.712
Cl, μmol/g	19.08	20.89	22.41	22.96	0.908	0.155	0.525	0.731
Mg, μmol/g	10.85	10.04	10.84	10.65	0.462	0.765	0.615	0.755
P, μmol/g	20.01	21.07	17.18	17.72	0.702	0.030	0.553	0.848
Descending colon								
Water, %	72.45	73.91	76.31	75.82	0.505	0.002	0.566	0.255
Na, mmol/g	0.090	0.101	0.105	0.104	0.0027	0.075	0.339	0.279
K, mmol/g	0.057	0.051	0.045	0.050	0.0024	0.202	0.891	0.239
Ca, μmol/g	2.85	2.83	4.22	4.24	0.265	0.009	0.996	0.960
Fe, μmol/g	0.103	0.117	0.152	0.154	0.007	0.001	0.450	0.590
Cl, μmol/g	19.80	22.94	25.31	25.71	1.009	0.041	0.361	0.477
Mg, μmol/g	12.18	10.78	9.63	12.22	0.578	0.632	0.612	0.096
P, μmol/g	22.64	24.00	18.44	20.84	0.819	0.023	0.222	0.730

LC—lignocellulose, PF—potato fiber, ZnGly—zinc chelate of glycine hydrate.

**Table 2:** Histological parameters and mucus layer thickness in the cecum and colon of pigs.

Parameter	LC		PF		SEM	P		
	ZnSO_4_	ZnGly	ZnSO_4_	ZnGly		Fiber	Zn	Interaction
Cecum								
Crypt depth, μm	585	577	522	548	12.1	0.063	0.700	0.475
Muscular layer thickness, μm	903	908	1014	955	38.1	0.331	0.736	0.690
IEL/100 epithelial cells	6.0	5.1	4.9	5.6	0.35	0.680	0.915	0.312
Mucus layer thickness, μg dye/cm^2^	73.62	71.49	81.78	74.61	3.320	0.423	0.509	0.7191
Ascending colon								
Crypt depth, μm	564	581	534	503	15.5	0.094	0.821	0.446
Muscular layer thickness, μm	639	610	661	686	52.3	0.664	0.987	0.812
IEL/100 epithelial cells	9.0	8.2	8.7	9.3	0.30	0.552	0.903	0.282
Mucus layer thickness, μg dye/cm^2^	67.71	70.49	76.52	78.16	4.062	0.344	0.798	0.947
Transverse colon								
Crypt depth, μm	610	566	605	561	13.5	0.849	0.119	0.999
Muscular layer thickness, μm	601	685	717	642	29.4	0.552	0.942	0.201
IEL/100 epithelial cells	6.6	6.1	7.3	5.9	0.43	0.786	0.290	0.639
Mucus layer thickness, μg dye/cm^2^	60.61	62.58	75.31	71.96	3.691	0.120	0.927	0.724
Descending colon								
Crypt depth, μm	721	699	667	665	15.1	0.164	0.705	0.750
Muscular layer thickness, μm	688	675	742	697	26.2	0.497	0.608	0.771
IEL/100 epithelial cells	4.1	3.2	3.3	3.8	0.28	0.839	0.701	0.247
Mucus layer thickness, μg dye/cm^2^	62.28	74.44	65.2	73.09	3.319	0.908	0.152	0.755

LC—lignocellulose, PF—potato fiber, ZnGly—zinc chelate of glycine hydrate, IEL—intraepithelial lymphocytes.

## Data Availability

Raw data are available from the corresponding author upon reasonable request.
